# The Contact Dermatitis Quality of Life Index (CDQL): Survey Development and Content Validity Assessment

**DOI:** 10.2196/30620

**Published:** 2021-12-16

**Authors:** Mary K Hill, Melissa R Laughter, Cecile I Harmange, Robert P Dellavalle, Chandler W Rundle, Cory A Dunnick

**Affiliations:** 1 Department of Dermatology University of Colorado Anschutz Medical Campus Aurora, CO United States; 2 School of Medicine University of Colorado Aurora, CO United States; 3 Dermatology Service United States Department of Veterans Affairs Eastern Colorado Health Care System Aurora, CO United States; 4 Department of Epidemiology Colorado School of Public Health University of Colorado Anschutz Medical Campus Aurora, CO United States; 5 School of Medicine Loma Linda University Loma Linda, CA United States

**Keywords:** contact dermatitis, allergic contact dermatitis, irritant contact dermatitis, quality of life, outcomes instruments, health outcomes

## Abstract

**Background:**

There is limited measurement and reporting of quality of life (QoL) outcomes for patients with contact dermatitis (CD).

**Objective:**

The purpose of this study is to develop a standardized Contact Dermatitis Quality of Life index (CDQL) for adult patients.

**Methods:**

A list of 81 topics was compiled from a review of QoL measures used previously in CD research. A total of 2 rounds of web-based Delphi surveys were sent to physicians who registered to attend the 2018 American Contact Dermatitis Society meeting, asking that they rank the relevance of topics for measuring QoL in CD using a 4-point scale. Items met consensus for inclusion if at least 78% of respondents ranked them as relevant or very relevant, and their median score was ≥3.25.

**Results:**

Of the 210 physicians contacted, 34 physicians completed the initial survey and 17 completed the follow-up survey. A total of 22 topics met consensus for inclusion in the CDQL, addressing symptoms, emotions, functions of daily living, social and physical functions, work/school functions, and treatment.

**Conclusions:**

This study was limited by the following factors: few open-ended questions in the initial survey, a lack of direct patient feedback, and long survey length, which likely contributed to lower survey participation. The CDQL is a comprehensive, CD-specific QoL measure developed on the basis of expert consensus via a modified Delphi process to be used by physicians and other health care professionals who care for adult patients with contact dermatitis.

## Introduction

Measures of quality of life (QoL) have become a fundamental component in evaluating the benefits of dermatologic interventions, especially for chronic, incurable diseases. Supplementary to the objective clinical indices used to assess disease severity, QoL instruments incorporate patients’ impressions of their functioning and well-being, allowing for a more complete picture of their health status. Unlike generic questionnaires, disease-specific instruments are more responsive to changes over time in QoL [1,2].

The negative impact of contact dermatitis (CD) on QoL has been established in existing literature [3-13]. Worse QoL is associated with the presence of several features, including pruritus, discomfort, and trouble working with one’s hands or carrying out everyday activities [14]. Chronically, the impact of dermatologic diseases on QoL can result in considerable emotional and functional impairment [15]. The extent of CD’s effect on QoL is not always adequately reflected by disease severity, possibly due to the psychological stress and embarrassment caused by visual manifestations of the disease [14]. It is therefore essential to use a standardized tool for quantitatively assessing QoL in patients with CD. However, as revealed by a systematic review of outcomes instruments used for CD in randomized controlled trials (RCTs) published between 2005 and 2015 [16], only a small minority of RCTs (6%) assessed QoL, and among those studies, there was a lack of consensus on what tool to use for this purpose.

A standardized measure of QoL for adult patients with CD would be beneficial in guiding individual treatment strategies and to potentially help prevent the risks associated with chronically depressed QoL. Additionally, such a universal tool would allow for greater comparability among articles in the CD literature. The purpose of this study, therefore, was to develop the Contact Dermatitis Quality of Life index (CDQL), a QoL measure specific to CD that quantifies the impact of the disease on functioning and well-being from a patient perspective. This tool was created for use by physicians and other health care professionals caring for patients with contact dermatitis.

## Methods

The process of developing the CDQL consisted of initial topic generation via a literature review, followed by a 2-step modified Delphi method to establish the content validity of the instrument.

Preliminary topics compiled for the questionnaire were based on a review of QoL outcome measures used in previous studies of CD. A systematic review [16] of CD outcome measures in RCTs published from 2005 to 2015 found that QoL was evaluated using the Dermatology Life Quality Index (DLQI) [17] and various general assessments of pruritus. According to a 2003 literature review [12], other QoL tools used for patients with CD include the Dermatology-Specific Quality of Life (DSQL) instrument [15], the Skindex-29 [18,19], and the 36-item Short Form Health Survey [20]. Additionally, the Skindex-16 [21] was previously modified for use in allergic CD, with the addition of 5 questions specific to the effect on one’s occupation [22]. A subsequent QoL measure for CD incorporated modifications of both the Skindex-16 and the DLQI, as well as 6 additional items addressing feelings and functioning [14].

A total of 81 topics were generated from a review of the aforementioned QoL instruments. Similar to the Skindex-16 [21], each topic was worded to ask patients how often the event in the topic bothered them. Expert consensus was sought regarding questionnaire topics in accordance with a modified Delphi technique, with 2 rounds of surveys conducted to maximize consensus [23]. Following institutional review board approval, the initial voluntary, anonymous web-based surveys were sent to the 210 registrants of the 2018 annual meeting of the American Contact Dermatitis Society (ACDS), asking that dermatology physicians rank the relevance of each questionnaire topic using the following 4-point Likert scale: (1) not relevant, (2) somewhat relevant, (3) relevant, or (4) very relevant (Multimedia Appendix 1). Topics derived from the Skindex-16 were italicized. Survey respondents were also asked to provide their opinion regarding the time frame which the CDQL should be designed to address, keeping in mind both the potentially intermittent nature of CD [15] and the goal of maximizing patient recollection [17].

Definitions of consensus vary throughout the literature. A prior systematic review investigating consensus in Delphi studies found that consensus is most often defined by the percentage of agreement, followed by the proportion of subjects’ ratings falling within a specified range [24]. Thresholds set for consensus definitions based on percentages or proportions range from 50% to 97%, with a median of 75%. Green et al [25,26] suggested that consensus is achieved when at least 70% of Delphi respondents rank the item as 3 or 4 on a 4-point Likert scale, and the median is at least 3.25. Lynn et al [27,28] suggested that with at least 6 professionals ranking the relevance of a topic for a new instrument, the content validity index (CVI) of the topic (the proportion of professionals ranking it as a 3 or 4 on a 4-point scale) should be ≥0.78 in order to reduce the possibility of agreement due to chance. A combination of these criteria was used for this study, with items meeting consensus for inclusion in the CDQL if at least 78% of respondents ranked them as relevant or very relevant (a score of 3 or 4), and the median score was at least 3.25. Also in line with precedent [29-34], items rated as relevant or very relevant by less than 50% of respondents were excluded.

In response to expert comments from the initial survey recommending less repetition and a shorter questionnaire length to improve practicality for clinical use, similar questionnaire topics were combined and/or excluded. The remaining topics with CVIs of 50% to 77% were compiled in a second survey, which listed the initial CVI for each item and asked respondents of the first survey to rank topic relevance on a 4-point scale again (Multimedia Appendix 2). A total of 7 new topics were included in the second survey based on preliminary results from a study aimed at developing a QoL index for allergic CD [35]. Additionally, based on comments from the initial survey, 4 other new topics were included under a treatment domain. Survey respondents were asked to provide a brief explanation for their ranking of relevance if the initial CVI for a topic was <60% or if they ranked an item with a CVI of >60% as somewhat relevant or not relevant. Again, individual items from the second survey were included as items in the final CDQL if the CVI among respondents was ≥0.78 and the median score was ≥3.25.

In order to further establish the CDQL’s content validity, the CVI for the total scale was calculated. Different ways of quantifying this value exist, although it is recommended (especially when larger numbers of experts are involved, as in this study) that it be calculated by taking the average of the CVIs for the individual questionnaire topics [28]. A total scale CVI of ≥0.90 has been previously deemed acceptable [28,36].

Survey data were collected and managed using REDCap (Research Electronic Data Capture; Vanderbilt University) electronic data capture tools hosted at the University of Colorado Denver [37]. REDCap is a secure, web-based application designed to support data capture for research studies, providing the following: (1) an intuitive interface for validated data entry, (2) audit trails for tracking data manipulation and export procedures, (3) automated export procedures for seamless data downloads to common statistical packages, and (4) procedures for importing data from external sources. Statistical analysis was performed using Excel (Microsoft Corporation).

This study was reviewed and approved by The Colorado Multiple Institutional Review Board.

## Results

Of the 210 individuals contacted, 43 (20.5%) completed the initial Delphi survey. A total of 8 surveys were completed by nonphysicians and were therefore excluded. Additionally, 13 partially completed surveys were excluded. A total of 34 physicians completed the initial survey, of whom 33 were attending dermatologists and 1 was a fellow. Of the 34 physicians who completed the survey, 27 (79%) patch tested >41 patients per year; only 1 physician did not do any patch testing. All but 2 of the physicians were members of the ACDS, American Academy of Dermatology, and/or American Academy of Allergy, Asthma, and Immunology.

A total of 12 topics from the first Delphi survey with a CVI of <50% were excluded. Of the remaining topics, 22 were deemed repetitive and also excluded. Initially, 23 topics from the first Delphi survey met consensus for inclusion in the CDQL; however, based on expert feedback, several redundant topics were either removed or combined, ultimately resulting in 19 topics meeting consensus for inclusion (Table 1). The CVIs and median relevance scores for these topics ranged from 0.79 to 1.0 and 3.5 to 4.0, respectively.

The follow-up Delphi survey consisted of 35 questionnaire topics not yet meeting consensus for inclusion or exclusion (including 11 new topics). Of the 43 individuals contacted (those who had responded to the initial survey), 23 (53%) completed the second Delphi survey. The final analysis included a total of 17 surveys fully completed by physicians who had also completed the initial survey. Following completion of the second survey, an additional 4 topics met consensus for inclusion, with CVIs ranging from 0.82 to 1.0, and a median relevance score of 4.0 for all 4 questions (Table 2).

Based on the first survey, 20 (59%) of the 34 respondents felt the questionnaire should ask about QoL over the past 6 months, 8 (24%) felt it should address the past month, 4 (12%) felt it should address the past year, and 2 (6%) felt it should address the past week. Agreement improved in the follow-up survey, with 16 (70%) of the 23 respondents suggesting that the CDQL inquire about the past 6 months.

The resulting CDQL consists of 23 items, asking patients how often they have been bothered by each item over the past 6 months (Multimedia Appendix 3). Responses are structured on a 4-point Likert scale: (1) never bothered, (2) sometimes bothered, (3) often bothered, or (4) always bothered. For ease of use, this was simplified from the Skindex-16 [21], which uses a continuous bipolar scale with 7 answer choices.

The CDQL can be broken down into 6 different domains: symptoms (1 item), emotions (9 items), functions of daily living (3 items), social and physical functions (2 items), work/school functions (4 items), and treatment-related items (4 items). The CVI for the total scale was 0.85. A total of 10 topics were at least in part derived from the Skindex-16 [21], 9 topics were derived from the Skindex-29 [18,19], 7 topics were derived from the DSQL [15], 5 topics were derived from the DLQI [17], 6 topics were derived from the CD-specific quality of life measure by Ayala et al [14], 2 topics were derived from the 36-item Short Form Health Survey [20], 2 topics were derived from the modified Skindex-16 by Kadyk et al [22], and 3 topics were based on expert recommendations from the first survey.

**Table 1:** Topics meeting consensus for inclusion after the initial Delphi survey.

Topics^a^		Content validity index^b^	Median (SD)^c^
**Symptoms**			
	Itching of your skin^d,e,f,g^	1.0	4.0 (0.24)
**Emotions**			
	Your skin condition persisting or reoccurring^d^	0.94	4.0 (0.58)
	Your skin condition's appearance^d^	0.91	4.0 (0.65)
	Frustration because of your skin condition^d,e,f^	0.91	4.0 (0.66)
	Feeling embarrassed^d,e,f,g^ or ashamed^e^ because of your skin condition^h^	0.82-0.91	3.5-4.0 (0.66-0.84)
	Feeling uncomfortable because of your skin condition^i^	0.91	4.0 (0.75)
	Feeling annoyed or irritated because of your skin condition^d,e,^^i^	0.85	3.5 (0.87)
	Feeling depressed because of your skin condition^d,e^	0.85	4.0 (0.82)
	Lack of self-confidence because of your skin condition^f^	0.82	4.0 (0.85)
	Concern about what others think about you because of your skin condition^f^	0.82	4.0 (0.86)
**Functions of daily living**			
	Effects of your skin condition on your daily activities^d^	0.97	4.0 (0.38)
	Your skin condition interfering with your sleep^e,i^	0.97	4.0 (0.53)
**Social and physical functions**			
	Effects of your skin condition on your social or leisure activities^e,f,g,k^	0.88	4.0 (0.70)
	Effects of your skin condition on your interactions with others (for example, your partner, friends, or relatives)^d,e^	0.85	4.0 (0.81)
**Work/school functions**			
	Difficulties using your hands at work because of your skin condition^i,j^	0.91	4.0 (0.75)
	Difficulties working or studying because of your skin condition^d,e,g,k^	0.85	4.0 (0.75)
	Concerns that you may lose your job (either because you need to quit or are fired) due to your skin condition^i,j,l^	0.85	4.0 (0.82-0.83)
	Effects of your skin condition on your finances^i^	0.79	4.0 (0.94)
**Treatment**			
	Problems from the treatment of your skin condition (for example, taking up time or being messy)^g,m^	0.88	3.5 (0.77)

^a^Topics are intended to ask patients how often they have been bothered by them.

^b^The proportion of physicians ranking a topic’s relevance as 3 or 4 on a 4-point Likert scale: (1) not relevant, (2) somewhat relevant, (3) relevant, or (4) very relevant. All values are based on a total of 34 physicians completing the survey.

^c^Values are based on relevance scoring using a 4-point scale, as noted previously.

^d^Topics derived from the Skindex-16 [21].

^e^Topics derived from the Skindex-29 [18,19].

^f^Topics derived from the Dermatology-Specific Quality of Life (DSQL) instrument [15].

^g^Topics derived from the Dermatology Life Quality Index (DLQI) [17].

^h^The following topics were combined: “embarrassment because of your skin condition” and “feeling ashamed of your skin condition.” Listed values display the range of values for the combined topics.

^i^Topics derived from a contact dermatitis (CD)-specific quality of life measure from Ayala et al [14].

^j^Topics derived from the 36-item Short Form Health Survey [20].

^k^Topics derived from a modified Skindex-16 from Kadyk et al for use in allergic CD [22].

^l^The following topics were combined: “concerns that you may need to quit your job because of your skin condition” and “concerns about being fired from your job because of your skin condition.” The range of standard deviations is listed; other values for the two combined topics were the same.

^m^Prior to the development of a treatment domain in the second round of surveying, this topic was initially categorized under functions of daily living.

**Table 2:** Topics meeting consensus for inclusion after the second Delphi survey.

Topics^a^		Content validity index^b^	Median (SD)^c^	
**Functions of daily living**				
	Limitations in shaving or wearing makeup because of your skin condition^d^	0.88		4.0 (0.86)
**Treatment**				
	Lack of treatment success using recommended remedies for your skin condition^e^	1		4.0 (0.51)
	Difficulty finding products that are safe for your skin^e^	0.94		4.0 (0.62)
	The cost of products that are safe for your skin^e^	0.82		4.0 (0.93)

^a^Topics are intended to ask patients how often they have been bothered by them.

^b^The proportion of physicians ranking a topic’s relevance as 3 or 4 on a 4-point Likert scale: (1) not relevant, (2) somewhat relevant, (3) relevant, or (4) very relevant. All values are based on a total of 34 physicians completing the survey.

^c^Values are based on relevance scoring using a 4-point scale, as noted previously.

^d^Topics derived from the Dermatology-Specific Quality of Life (DSQL) instrument [15].

^e^Topics added to the second survey round based on expert recommendations from the first survey.

## Discussion

There are multiple tools to assess QoL in dermatology; however, few of these tools have been validated for use in CD. The 36-item Short Form Health Survey is frequently used in dermatology as a broad questionnaire to assess a wide variety of skin concerns. The DLQI, DSQL instrument for CD, Skindex-16 and its modified versions, and Skindex-29 are more commonly used tools for measuring QoL specifically in CD [38]. However, there are many aspects important for assessing QoL that are not completely incorporated into these questionnaires [39]. Some areas lacking in these questionnaires include psychosocial impact, impact on occupation, and treatment concerns. For these reasons, we developed a new QoL tool specific to CD that can adequately assess all important aspects of QoL in one complete questionnaire. This tool aims to increase detection of QoL changes related to CD in order to better assess disease-related QoL, disease progression, and response to therapies.

Previously validated tools such as the Skindex-16 and the Skindex-29 were used to aid the creation of our new tool. Topics such as those exploring stinging or burning of the skin, irritation of the skin, and worry caused by the skin condition are all validated questions present in the Skindex-16 and also included in the CDQL; these overlapping topics are indicated in Table 1. In terms of more recently published QoL measures, the disease-specific questionnaire for allergic contact dermatitis proposed by Botto et al [35] explores a variety of topics that are also included in the CDQL, such as “concern for infecting others because of your skin condition” and “I am bothered by cracking of my skin.” While the CDQL includes similar types of questions under the categories of function, emotions, and symptoms, it also further addresses topics of “functions of daily living” and “work and school function,” allowing for a more complete understanding of the impact this skin condition has on patients’ daily lives. For example, we include impacts on types of clothes worn, the ability to participate in certain sports, and the duration of time needed to find treatment or care for their condition. Additionally, our tool examines contact dermatitis more broadly, rather than focusing on the specific subset of allergic contact dermatitis, allowing for a more universal application of the tool.

The Delphi technique, a series of successive questionnaires aimed at determining opinion consensus among a group of experts [40], was used to formulate the CDQL. The strength of this technique comes from its ability to efficiently achieve consensus on topics of uncertainty [41]. Furthermore, the controlled feedback following each round of the questionnaire can broaden thinking and stimulate new ideas among experts [42]. However, the weakness of the Delphi technique typically stems from a lack of agreement on how consensus is defined [43]. Varying interpretations and methodology to define consensus and validity can diminish the credibility of this technique.

The precedent is to deem the content validity of an instrument excellent if the following criteria are met: (1) The CVIs for individual topics are ≥0.78 when at least 6 experts are assessing the relevance of the topics, and (2) the CVI of the total scale (when calculated in the same manner as for this study) is ≥0.90 [27,28,36]. The final individual topics included in this tool had CVIs ranging from 0.79 to 1.0. However, the CVI of the total scale was 0.85, falling slightly below the previously determined 0.90 standard to be considered excellent. Of note, some studies recommend a minimum total scale CVI of 0.80 [44]. While this may be a more realistic benchmark for the total CVI, some researchers have argued that a total scale CVI of 0.90 would better protect against exceedingly low individual CVIs (eg, <0.4) [36]. As the CDQL had final individual topic CVIs ranging from 0.79 to 1.0, a total scale CVI ≥0.80 may be a suitable indication of content validity.

One limitation of this study was the long length of the surveys, which likely played a role in the lower survey completion rate. Additionally, while a typical Delphi method would have consisted of an initial survey with a series of open-ended questions intended to generate a list of QoL issues [45], this was replaced by a literature search in this study. Nevertheless, experts were still given the opportunity in the first survey to note additional topics that they felt were relevant. Additionally, while this study did not directly incorporate patient feedback during development of the scale, the second round of surveying incorporated unique topics from another recent study [35] aimed at developing a QoL index for allergic CD based on patient interviews. This index is intended for use in conjunction with another more comprehensive QoL scale, whereas the CDQL is designed to be sufficient by itself for assessing QoL in CD. Furthermore, 1 respondent to the initial survey felt that the questionnaire was limited by its lack of items incorporating intensity and localization of CD. However, these factors are specific to disease severity and the CDQL is meant to be used in combination with, not in lieu of, a validated disease severity tool. As previously noted, the degree of impact of CD on QoL may not always correlate with disease severity [14].

Future studies are planned to further establish the CDQL’s validity, reliability, and responsiveness to changes in QoL. It is hoped that the resulting validated outcomes instrument will be suitable for use in both clinical practice and research to quantitatively determine the effect of health care interventions on QoL among patients with CD.

### Funding Sources

None.

## Appendix

Multimedia Appendix 1 Initial questionnaire sent to registrants of the 2018 American Contact Dermatitis Society meeting.

Multimedia Appendix 2 Second round of the questionnaire sent out to respondents.

Multimedia Appendix 3 Contact Dermatitis Quality of Life Index.

## Abbreviations

ACDS: American Contact Dermatitis Society
CD: contact dermatitis
CDQL: Contact Dermatitis Quality of Life index
CVI: content validity index
DLQI: Dermatology Life Quality Index
DSQL: Dermatology-Specific Quality of Life instrument
NIH/NCRR CSTI: National Institutes of Health/National Center for Research Resources Colorado Clinical and Translational Science Institute
QoL: quality of life
RCT: randomized controlled trial
REDCap: Research Electronic Data Capture
