# Supplementary material for: The Contact Dermatitis Quality of Life Index (CDQL): Survey Development and Content Validity Assessment
Source: JMIR Dermatol. 2021 Dec 16;4(2):e30620. doi: 10.2196/30620 (PMC10334971; doi:10.2196/30620)
Supplement: Multimedia Appendix 1 [file derma_v4i2e30620_app1.docx]

**Multimedia Appendix 1. Initial Survey.**

What is your current position at your practice/institution?

- Attending dermatologist
- Fellow
- Resident
- Intern
- Not a physician

On average, how may patients do you patch test in a year?

- >41
- 21-40
- 1-20
- None

Which of the following societies are you a member of? (Check as many as apply)

- American Contact Dermatitis Society
- American Academy of Dermatology
- American Academy of Allergy, Asthma, and Immunology
- None of the above

We intend to develop and/or validate a new quality of life measure specific to contact dermatitis. The following topics were derived from various quality of life measures used in previous studies of contact dermatitis. Based on your expert opinion, please rate the relevance of each of the following items for assessing quality of life in adult patients with contact dermatitis. Please note that these items are designed to ask how often patients have been bothered by them. Questions derived from the Skindex-16 are italicized.

|  |  | Not Relevant | Somewhat Relevant | Relevant | Very Relevant |
| --- | --- | --- | --- | --- | --- |
| **Symptoms:** | |  |  |  |  |
|  | *Itching of your skin* |  |  |  |  |
|  | *Stinging or burning of your skin condition* |  |  |  |  |
|  | *Your skin condition hurting* |  |  |  |  |
|  | *Irritation of your skin* |  |  |  |  |
|  | Water bothering your skin condition |  |  |  |  |
|  | Soreness or tenderness of your skin condition |  |  |  |  |
|  | Sensitivity of your skin |  |  |  |  |
|  | Bleeding of your skin condition |  |  |  |  |
|  | Dryness of your skin condition |  |  |  |  |
| **Emotions:** | |  |  |  |  |
|  | *Your skin condition persisting or reoccurring* |  |  |  |  |

|  | *Worrying about your skin condition (for example, that it will worsen, scar, act unpredictably, etc.)* |  |  |  |  |
| --- | --- | --- | --- | --- | --- |
|  | *Your skin condition’s appearance* |  |  |  |  |
|  | *Frustration because of your skin condition* |  |  |  |  |
|  | *Embarrassment because of your skin condition* |  |  |  |  |
|  | *Feeling annoyed or irritated because of your skin condition* |  |  |  |  |
|  | Anger about your skin condition |  |  |  |  |
|  | *Feeling depressed because of your skin condition* |  |  |  |  |
|  | Feeling ashamed of your skin condition |  |  |  |  |
|  | Feeling uncomfortable because of your skin condition |  |  |  |  |
|  | Concern about infecting others because of your skin condition |  |  |  |  |
|  | Lack of self-confidence because of your skin condition |  |  |  |  |
|  | Concern about what others think about you because of your skin condition |  |  |  |  |
| **Functions of Daily Living:** | |  |  |  |  |
|  | *Effects of your skin condition on your daily activities* |  |  |  |  |
|  | Your skin condition interfering with you going shopping |  |  |  |  |
|  | Your skin condition interfering with you looking after your home |  |  |  |  |
|  | Your skin condition interfering with you looking after your garden |  |  |  |  |
|  | Your skin condition influencing the clothes you wear |  |  |  |  |
|  | Your skin condition interfering with your sex life |  |  |  |  |

|  | Problems from the treatment of your skin condition (for example, taking up time or being messy) |  |  |  |  |
| --- | --- | --- | --- | --- | --- |
|  | Your skin condition interfering with your sleep |  |  |  |  |
|  | Limitations in shaving or wearing makeup because of your skin condition |  |  |  |  |
|  | Limitations in your choice of hairstyle because of your skin condition |  |  |  |  |
|  | Limitations in your food/beverage choices because of your skin condition |  |  |  |  |
|  | Difficulties performing your normal daily activities because of physical problems caused by your skin condition |  |  |  |  |
|  | Less time spent on your normal daily activities because of physical problems caused by your skin condition |  |  |  |  |

|  | Accomplishing less than you would like in your normal daily activities because of physical problems caused by your skin condition |  |  |  |  |
| --- | --- | --- | --- | --- | --- |
|  | Limitations in the kinds of daily activities you are able to do because of physical problems caused by your skin condition |  |  |  |  |
|  | Less time spent on your normal daily activities because of emotional problems caused by your skin condition (for example, because of feeling depressed or embarrassed) |  |  |  |  |
|  | Accomplishing less than you would like in your normal daily activities because of emotional problems caused by your skin condition (for example, because of feeling depressed or embarrassed) |  |  |  |  |

|  | Not doing your normal daily activities as carefully as usual because of emotional problems caused by your skin condition (for example, because of feeling depressed or embarrassed) |  |  |  |  |
| --- | --- | --- | --- | --- | --- |
| **Social and Physical Functions:** | |  |  |  |  |
|  | Effects of your skin condition on your social or leisure activities |  |  |  |  |
|  | *Your skin condition making it difficult to do what you enjoy* |  |  |  |  |
|  | *Effects of your skin condition on your interactions with others (for example, your partner, friends, or relatives)* |  |  |  |  |
|  | *Effects of your skin condition on your desire to be around people* |  |  |  |  |
|  | Limited desire to date because of your skin condition |  |  |  |  |
|  | Feeling limited in your satisfaction with personal relationships because of your skin condition |  |  |  |  |
|  | Feeling limited in your chances for making friends because of your skin condition |  |  |  |  |
|  | *Difficulties showing affection because of your skin condition* |  |  |  |  |
|  | Tendency to stay at home because of your skin condition |  |  |  |  |
|  | Interference in your dating habits/plans because of your skin condition |  |  |  |  |
|  | Limited time spent in the community because of your skin condition |  |  |  |  |
|  | Feeling tired because of your skin condition |  |  |  |  |
|  | Your skin condition making it hard to do any sports |  |  |  |  |
|  | Your skin condition limiting your participation in vigorous physical activities (for example, running or other strenuous sports) |  |  |  |  |
|  | Your skin condition limiting your participation in moderate physical activities (for example, playing golf or bowling) |  |  |  |  |
|  | Limitations in lifting or carrying groceries because of your skin condition |  |  |  |  |
|  | Limitations in climbing several flights of stairs because of your skin condition |  |  |  |  |
|  | Limitations in climbing one flight of stairs because of your skin condition |  |  |  |  |
|  | Limitations in bending, kneeling, or stooping because of your skin condition |  |  |  |  |
|  | Limitations in walking more than a mile because of your skin condition |  |  |  |  |
|  | Limitations in walking several blocks because of your skin condition |  |  |  |  |
|  | Limitations in walking one block because of your skin condition |  |  |  |  |

|  | Limitations in bathing or dressing yourself because of your skin condition |  |  |  |  |
| --- | --- | --- | --- | --- | --- |
| **Work/School Functions:** | |  |  |  |  |
|  | *Difficulties working or studying because of your skin condition* |  |  |  |  |
|  | Concerns that you may need to quit your job because of your skin condition |  |  |  |  |
|  | Effects of your skin condition on your finances |  |  |  |  |
|  | Difficulties interacting with coworkers or classmates because of your skin condition |  |  |  |  |
|  | Difficulties using your hands at work because of your skin condition |  |  |  |  |
|  | Concerns about being fired from your job because of your skin condition |  |  |  |  |
|  | Limitations in the kind of work you are able to do because of physical problems caused by your skin condition |  |  |  |  |
|  | Less time spent on work or school because of physical problems caused by your skin condition |  |  |  |  |
|  | Less time spent on work or school because of emotional problems caused by your skin condition (for example, because of feeling depressed or embarrassed) |  |  |  |  |
|  | Accomplishing less than you would like at work or school because of physical problems caused by your skin condition |  |  |  |  |
|  | Accomplishing less than you would like at work or school because of emotional problems caused by your skin condition (for example, because of feeling depressed or embarrassed) |  |  |  |  |

|  | Not doing your work or schoolwork as carefully as usual because of emotional problems caused by your skin condition (for example, because of feeling depressed or embarrassed) |  |  |  |  |
| --- | --- | --- | --- | --- | --- |
|  | Difficulties getting ahead at work or school because of your skin condition |  |  |  |  |
|  | Difficulties getting a better job because of your skin condition |  |  |  |  |
|  | Difficulties being effective in meetings because of your skin condition |  |  |  |  |
|  | Difficulties being effective in giving directions because of your skin condition |  |  |  |  |
|  | Difficulties being punctual due to doctors’ appointments for your skin condition |  |  |  |  |
|  | Difficulties being punctual due to physical or emotional discomfort from your skin condition |  |  |  |  |

Are there any other topics not previously addressed that you think should be included in a quality of life measure for adult patients with contact dermatitis?

_____________________________________________________________________________________

_____________________________________________________________________________________

_____________________________________________________________________________________

Based on your expert opinion, over what time frame should the quality of life questionnaire be designed to address? Factors to consider include the potentially intermittent presence of contact dermatitis and desire to maximize patient recollection.

- Over the past week
- Over the past month
- Over the past six months
- Over the past year
