# Supplementary material for: The Contact Dermatitis Quality of Life Index (CDQL): Survey Development and Content Validity Assessment
Source: JMIR Dermatol. 2021 Dec 16;4(2):e30620. doi: 10.2196/30620 (PMC10334971; doi:10.2196/30620)
Supplement: Multimedia Appendix 2 [file derma_v4i2e30620_app2.docx]

**Multimedia Appendix 2. Second Survey.**

| **INSTRUCTIONS**  **° Based on your expert opinion, please rate the relevance of each of the following items for assessing quality of life in adult patients with contact dermatitis**  **° Please recall these items are designed to ask how often patients have been bothered by them**  **° Questions derived from the Skindex-16 are italicized** | | | | | | |
| --- | --- | --- | --- | --- | --- | --- |
| **SYMPTOMS** | |  |  |  | |  |
| **Topics meeting consensus for inclusion in our quality of life measure thus far (based on the first survey results):** | | | | | | |
| **1. Itching of your skin** | | | | | | |
|  | | | | | | |
|  | | Not Relevant | Somewhat Relevant | | Relevant | Very Relevant |
|  | *Stinging or burning of your skin condition*  Note: In the last survey round, 76% of respondents found this relevant or very relevant. |  |  | |  |  |
|  | [Conditional question if answered not relevant or somewhat relevant] Please provide a brief explanation for your answer to the last topic. | | | | | |
|  | *Irritation of your skin*  Note: In the last survey round, 73% of respondents found this relevant or very relevant. |  |  |  | |  |
|  | [Conditional question if answered not relevant or somewhat relevant] Please provide a brief explanation for your answer to the last topic. | | | | | |
|  | Sensitivity of your skin  Note: In the last survey round, 68% of respondents found this relevant or very relevant. |  |  |  | |  |
|  | [Conditional question if answered not relevant or somewhat relevant] Please provide a brief explanation for your answer to the last topic. | | | | | |
|  | Dryness of your skin condition  Note: In the last survey round, 68% of respondents found this relevant or very relevant. |  |  |  | |  |
|  | [Conditional question if answered not relevant or somewhat relevant] Please provide a brief explanation for your answer to the last topic. | | | | | |

|  | Soreness or tenderness of your skin condition  Note: In the last survey round, 68% of respondents found this relevant or very relevant. |  |  |  |  |
| --- | --- | --- | --- | --- | --- |
|  | [Conditional question if answered not relevant or somewhat relevant] Please provide a brief explanation for your answer to the last topic. | | | | |
|  | Water bothering your skin condition  Note: In the last survey round, 65% of respondents found this relevant or very relevant. |  |  |  |  |
|  | [Conditional question if answered not relevant or somewhat relevant] Please provide a brief explanation for your answer to the last topic. | | | | |
|  | *Your skin condition hurting*  Note: In the last survey round, 65% of respondents found this relevant or very relevant. |  |  |  |  |
|  | [Conditional question if answered not relevant or somewhat relevant] Please provide a brief explanation for your answer to the last topic. | | | | |
|  | Cracking in your skin  Note: This topic was not included in the last survey round. |  |  |  |  |
|  | Sloughing and flaking from your skin condition  Note: This topic was not included in the last survey round. |  |  |  |  |
|  | Peeling from your skin condition  Note: This topic was not included in the last survey round. |  |  |  |  |

| **EMOTIONS**  **Topics meeting consensus for inclusion in our quality of life measure thus far (based on the first survey results):**  **1. Your skin condition persisting or reoccurring**  **2. Your skin condition's appearance**  **3. Frustration because of your skin condition**  **4. Feeling embarrassed or ashamed because of your skin condition**  **5. Feeling uncomfortable because of your skin condition**  **6. Feeling annoyed or irritated because of your skin condition**  **7. Feeling depressed because of your skin condition**  **8. Lack of self-confidence because of your skin condition**  **9. Concern about what others think about you because of your skin condition** | | | | | | | |
| --- | --- | --- | --- | --- | --- | --- | --- |
|  | | | | | | | |
|  | | Not Relevant | Somewhat Relevant | | | Relevant | Very Relevant |
|  | *Worrying about your skin condition (for example, that it will worsen, scar, act unpredictably, etc.)*  Note: In the last survey round, 73% of respondents found this relevant or very relevant. |  | |  | |  |  |
|  | [Conditional question if answered not relevant or somewhat relevant] Please provide a brief explanation for your answer to the last topic. | | | | | | |
|  | Concern about infecting others because of your skin condition  Note: In the last survey round, 56% of respondents found this relevant or very relevant. |  | |  |  | |  |
|  | Please provide a brief explanation for your answer to the last topic. | | | | | | |
|  | Feeling desperate because of your skin condition  Note: This topic was not included in the last survey round. |  | |  |  | |  |
|  | Feeling out of control because of your skin condition  Note: This topic was not included in the last survey round. |  | |  |  | |  |

| **FUNCTIONS OF DAILY LIVING**  **Topics meeting consensus for inclusion in our quality of life measure thus far (based on the first survey results):**  **1. Effects of your skin condition on your daily activities**  **2. Your skin condition interfering with your sleep** | | | | | | |
| --- | --- | --- | --- | --- | --- | --- |
|  | | | | | | |
|  | | Not Relevant | Somewhat Relevant | | Relevant | Very Relevant |
|  | Limitations in shaving or wearing makeup because of your skin condition  Note: In the last survey round, 88% of respondents found this relevant or very relevant. |  | |  |  |  |
|  | [Conditional question if answered not relevant or somewhat relevant] Please provide a brief explanation for your answer to the last topic. | | | | | |

|  | Your skin condition interfering with your sex life  Note: In the last survey round, 82% of respondents found this relevant or very relevant. |  |  |  |  |
| --- | --- | --- | --- | --- | --- |
|  | [Conditional question if answered not relevant or somewhat relevant] Please provide a brief explanation for your answer to the last topic. | | | | |
|  | Your skin condition influencing the clothes you wear  Note: In the last survey round, 76% of respondents found this relevant or very relevant. |  |  |  |  |
|  | [Conditional question if answered not relevant or somewhat relevant] Please provide a brief explanation for your answer to the last topic. | | | | |
|  | Limitations in your choice of hairstyle because of your skin condition  Note: In the last survey round, 68% of respondents found this relevant or very relevant. |  |  |  |  |
|  | [Conditional question if answered not relevant or somewhat relevant] Please provide a brief explanation for your answer to the last topic. | | | | |

|  | Limitations in your food/beverage choices because of your skin condition  Note: In the last survey round, 59% of respondents found this relevant or very relevant. |  |  |  |  |
| --- | --- | --- | --- | --- | --- |
|  | Please provide a brief explanation for your answer to the last topic. | | | | |
|  | Thinking about your skin condition all the time  Note: This topic was not included in the last survey round. |  |  |  |  |
|  | Difficulty concentrating or focusing because of your skin condition  Note: This topic was not included in the last survey round. |  |  |  |  |

| **SOCIAL AND PHYSICAL FUNCTIONS**  **Topics meeting consensus for inclusion in our quality of life measure thus far (based on the first survey results):**  **1. Effects of your skin condition on your social or leisure activities**  **2. Effects of your skin condition on your interactions with others (for example, your partner, friends, or relatives)** | | | | | | | |
| --- | --- | --- | --- | --- | --- | --- | --- |
|  | | | | | | | |
|  | | Not Relevant | Somewhat Relevant | | | Relevant | Very Relevant |
|  | *Your skin condition making it difficult to do what you enjoy*  Note: In the last survey round, 82% of respondents found this relevant or very relevant. |  | |  | |  |  |
|  | [Conditional question if answered not relevant or somewhat relevant] Please provide a brief explanation for your answer to the last topic. | | | | | | |
|  | *Effects of your skin condition on your desire to be around people*  Note: In the last survey round, 79% of respondents found this relevant or very relevant. |  | |  |  | |  |
|  | [Conditional question if answered not relevant or somewhat relevant] Please provide a brief explanation for your answer to the last topic. | | | | | | |
|  | Tendency to stay at home because of your skin condition  Note: In the last survey round, 76% of respondents found this relevant or very relevant. |  | |  |  | |  |
|  | [Conditional question if answered not relevant or somewhat relevant] Please provide a brief explanation for your answer to the last topic. | | | | | | |
|  | *Difficulties showing affection because of your skin condition*  Note: In the last survey round, 65% of respondents found this relevant or very relevant. |  | |  |  | |  |
|  | [Conditional question if answered not relevant or somewhat relevant] Please provide a brief explanation for your answer to the last topic. | | | | | | |

|  | Interference in your dating habits/plans because of your skin condition  Note: In the last survey round, 62% of respondents found this relevant or very relevant. |  |  |  |  |
| --- | --- | --- | --- | --- | --- |
|  | [Conditional question if answered not relevant or somewhat relevant] Please provide a brief explanation for your answer to the last topic. | | | | |
|  | Feeling limited in your satisfaction with personal relationships because of your skin condition  Note: In the last survey round, 56% of respondents found this relevant or very relevant. |  |  |  |  |
|  | Please provide a brief explanation for your answer to the last topic. | | | | |
|  | Limitations in bathing or dressing yourself because of your skin condition  Note: In the last survey round, 53% of respondents found this relevant or very relevant. |  |  |  |  |
|  | Please provide a brief explanation for your answer to the last topic. | | | | |

|  | Your skin condition making it hard to do certain sports  Note: This item is a combination of similar topics included in the last survey round. 53-62% of respondents found these topics relevant or very relevant. |  |  |  |  |
| --- | --- | --- | --- | --- | --- |
|  | Please provide a brief explanation for your answer to the last topic. | | | | |
|  | Limitations in lifting or carrying groceries because of your skin condition  Note: In the last survey round, 50% of respondents found this relevant or very relevant. |  |  |  |  |
|  | Please provide a brief explanation for your answer to the last topic. | | | | |

|  | Feeling limited in your chances for making friends because of your skin condition  Note: In the last survey round, 50% of respondents found this relevant or very relevant. |  |  |  |  |
| --- | --- | --- | --- | --- | --- |
|  | Please provide a brief explanation for your answer to the last topic. | | | | |
| **WORK/SCHOOL FUNCTIONS**  **Topics meeting consensus for inclusion in our quality of life measure thus far (based on the first survey results):**  **1. Difficulties using your hands at work because of your skin condition**  **2. Difficulties working or studying because of your skin condition**  **3. Concerns that you may lose your job (either because you need to quit or are fired) due to your skin condition**  **4. Effects of your skin condition on your finances**  **(There are no additional Work/School Function topics for you to rate.)**  **TREATMENT**  **Topics meeting consensus for inclusion in our quality of life measure thus far (based on the first survey results):**  **1. Problems from the treatment of your skin condition (for example, taking up time or being messy)** | | | | | |
|  | | | | | |

|  | | Not Relevant | Somewhat Relevant | | Relevant | Very Relevant |
| --- | --- | --- | --- | --- | --- | --- |
|  | Duration of time seeking medical care with no relief of your skin condition  Note: This topic was not included in the last survey round. |  | |  |  |  |
|  | Lack of treatment success using recommended remedies for your skin condition  Note: This topic was not included in the last survey round. |  | |  |  |  |
|  | Difficulty finding products that are safe for your skin  Note: This topic was not included in the last survey round. |  | |  |  |  |
|  | The cost of products that are safe for your skin  Note: This topic was not included in the last survey round. |  | |  |  |  |

Based on your expert opinion, over what time frame should the quality of life questionnaire be designed to address? Factors to consider include the potentially intermittent presence of contact dermatitis and desire to maximize patient recollection.

- Over the past week
- Over the past month
- Over the past six months
- Over the past year

Note: In the last survey round, 59% of respondents voted “over the past six months,” 23% voted “over the past month,” 12% voted “over the past year,” and 6% voted “over the past week.”

Please provide a brief explanation for your answer to the last question.

Any additional comments regarding the questionnaire?

_____________________________________________________________________________________

_____________________________________________________________________________________

_____________________________________________________________________________________
