# Supplementary material for: The Contact Dermatitis Quality of Life Index (CDQL): Survey Development and Content Validity Assessment
Source: JMIR Dermatol. 2021 Dec 16;4(2):e30620. doi: 10.2196/30620 (PMC10334971; doi:10.2196/30620)
Supplement: Multimedia Appendix 3 [file derma_v4i2e30620_app3.docx]

**Multimedia Appendix 3. Contact Dermatitis Quality of Life Index.**

How often have the following items related to your skin condition bothered you over the **past six months**? Please mark one box for each item.

| **Symptoms:** | | Never Bothered | Sometimes Bothered | Often Bothered | | Always Bothered |
| --- | --- | --- | --- | --- | --- | --- |
|  | Itching of your skin |  |  | |  |  |
| **Emotions:** | |  |  | |  |  |
|  | Your skin condition persisting or reoccurring |  |  | |  |  |

|  | Your skin condition’s appearance |  |  |  |  |
| --- | --- | --- | --- | --- | --- |
|  | Frustration because of your skin condition |  |  |  |  |
|  | Embarrassment because of your skin condition |  |  |  |  |
|  | Feeling uncomfortable because of your skin condition |  |  |  |  |
|  | Feeling annoyed or irritated because of your skin condition |  |  |  |  |
|  | Feeling depressed because of your skin condition |  |  |  |  |
|  | Lack of self-confidence because of your skin condition |  |  |  |  |
|  | Concern about what others think about you because of your skin condition |  |  |  |  |
| **Functions of Daily Living:** | |  |  |  |  |
|  | Effects of your skin condition on your daily activities |  |  |  |  |
|  | Your skin condition interfering with your sleep |  |  |  |  |
|  | Limitations in shaving or wearing makeup because of your skin condition |  |  |  |  |
| **Social and Physical Functions:** | |  |  |  |  |
|  | Effects of your skin condition on your social or leisure activities |  |  |  |  |
|  | Effects of your skin condition on your interactions with others (for example, your partner, friends, or relatives) |  |  |  |  |
| **Work/School Functions:** | |  |  |  |  |
|  | Difficulties working or studying because of your skin condition |  |  |  |  |
|  | Concerns that you may lose your job (either because you need to quit or are fired) due to your skin condition |  |  |  |  |
|  | Effects of your skin condition on your finances |  |  |  |  |
|  | Difficulties using your hands at work because of your skin condition |  |  |  |  |
| **Treatment:** | |  |  |  |  |
|  | Problems from the treatment of your skin condition (for example, taking up time or being messy) |  |  |  |  |

|  | Lack of treatment success using recommended remedies for your skin condition |  |  |  |  |
| --- | --- | --- | --- | --- | --- |
|  | Difficulty finding products that are safe for your skin |  |  |  |  |
|  | The cost of products that are safe for your skin |  |  |  |  |
